# Progress in Implementing National Policies and Strategies for Health Literacy—What Have We Learned so Far?

**DOI:** 10.3390/ijerph15071554

**Published:** 2018-07-23

**Authors:** Anita Trezona, Gill Rowlands, Don Nutbeam

**Affiliations:** 1 School of Health and Social Development, Deakin University, Melbourne 3125, Australia; 2 Institute of Health and Society, Newcastle University, Newcastle NE1 7RU, UK; Gill.Rowlands@newcastle.ac.uk; 3 School of Public Health, University of Sydney, Sydney 2006, Australia; don.nutbeam@sydney.edu.au

**Keywords:** health literacy, policy, policy analysis

## Abstract

Health literacy has been a prominent issue on the agenda of the World Health Organization (WHO) for almost two decades. WHO recently established a strong global mandate for public policy action on health literacy by positioning it as one of three key pillars for achieving sustainable development and health equity in the Shanghai Declaration on Health Promotion. Several countries have national health literacy policies, with many others expected to develop them in the immediate future. It is, therefore, timely to examine current policy approaches to health literacy. The purpose of this study was to analyze a selection of existing policy documents for their strengths, limitations and themes, and offer observations about their potential to improve health literacy and health outcomes. In doing so our intention is to offer lessons and advice from early adopters that will have usefulness for future policy development and implementation. We selected six policies for review; Australia, Austria, China, New Zealand, Scotland, and the United States. We used a set of criteria to guide a systematic analysis of policy documents for their context, intended target audiences, objectives, proposed actions and interventions, evidence of financial investment and intentions to monitor outcomes. We observed a number of common features that provide helpful signposting for future policy development in other countries. All represent a response to perceived deficiencies in the quality of patient communication and patient engagement. Most present health literacy as a universal challenge, with some also identifying groups who are of higher priority. They all recognize the importance of professional education in improving the quality of communication, and most recognize that the health literacy responsiveness of the health system needs to be improved. However, there was significant variability in linking resources to specific strategies and actions, as well as in the systems for monitoring progress and accountability for progress. This variability reflects important contextual differences between countries and health systems. However, this lack of specificity will likely have an impact on the priority given to improving health literacy and on the long-term sustainability of defined actions to improve health literacy in populations.

## 1. Introduction

Health literacy has become a subject of wide interest in the past decade, and several countries have current national health literacy policies or have incorporated health literacy as a priority issue within broader health strategies. It is not difficult to understand why. For researchers interested in health and disease causality, “health literacy” has become a convenient way of describing a measurable variable that can be used to understand and explain variation in health and disease outcomes. For clinicians, work over many years, mainly in the US, has established health literacy as an identifiable and manageable risk in clinical care, of particular importance in the management of long-term and complex conditions that depend upon successful patient engagement and management. For public health practitioners, health literacy is conceptually attractive in its fit with contemporary health promotion, understood as a personal “asset” that can be developed through educational and other interventions to support greater personal and community control over a range of determinants of health.

There is no doubt that wide scientific interest in health literacy has underpinned the policy interest. Over the past 20 years there has been a proliferation of studies that have provided definitions [1,2], conceptual models [3,4,5], and measurement tools [6,7,8]. Others have described the relationship between health literacy and health outcomes [9] and population prevalence [10,11]. This research has produced consistent evidence that low health literacy is a public health challenge across all parts of the world, and that it is modifiable through effective educational intervention and health system improvement.

This scientific interest has been mirrored by the growing interest of national and international organizations including, specifically, the World Health Organization (WHO) throughout this period. This was first evident from background work in support of a global conference on health promotion in Jakarta, Indonesia in 1997 [12] becoming a “key action” identified in the WHO Bangkok Charter for Health Promotion in a Globalized World [13]; and most recently described as one of three central strategies in the 2016 WHO Shanghai Declaration on promoting health in the 2030 Agenda for Sustainable Development [14].

The Shanghai Declaration has established a clear global mandate for the prioritization of health literacy within public policy, promoting the role and responsibility of governments to act. Countries and regions are already responding to this call to action [15], and the recently commissioned WHO Health Evidence Network synthesis report on Health Literacy Policy in the WHO European region [16], as well as the discussion generated through the first WHO Community of Practice on Health Literacy, provides evidence of the demand for guidance on effective health literacy policy development [17,18].

The policies and strategies that have emerged vary according to the way in which different countries and localities conceptualize health literacy and respond to perceived needs.

Each of the existing national policies and strategies has different origins, influences and processes that have informed their development and implementation, and have invariably been influenced by the political and health system contexts in which they were developed.

Policies articulate the intentions of governments to address public issues, including the priorities to be addressed and the courses of action to be taken [19]. These may take the form of broad informal policy statements, or more formalized documents such as policy frameworks, strategic plans or action plans. While health policies vary significantly in design and scope, most will or should contain common, observable elements. For example, they should establish a clear purpose, measurable goals and objectives, and actionable strategies [19]. They should also establish targets and specify mechanisms for monitoring, evaluating and reporting on implementation progress and policy outcomes [20]. This may include guidance to stakeholders on their roles and responsibilities in implementing the policy [21]. These elements can be objectively observed and reviewed for strengths and weaknesses, and general and specific lessons.

Policy responses to health literacy are likely to proliferate in the coming years, and it is timely to examine the early lessons emerging from those countries that have moved first in their public policy responses. The purpose of this study is to analyze a selection of existing policy documents for their strengths, limitations and themes, and offer observations about their potential to improve health literacy and health outcomes. In doing so our intention is to offer lessons and advice from early adopters that will have usefulness for future policy development and implementation.

## 2. Methods

Policy analysis is a retrospective, descriptive process of explaining various aspects of a particular policy. This may include the context, processes, actors and stakeholders (including their values and power), or the policy content such as the stated goals, intentions, actions and strategies [19,20,21]. Documentary analysis, or policy document analysis is a common approach to analyzing the content of policies, which can be useful for assessing the extent to which they contain the elements necessary to support successful implementation and achievement of the intended outcomes. Quantitative and qualitative methods can be used in policy document analysis [21]. Quantitative methods seek to systematically quantify the policy information according to predetermined categories, for example how many policies contain a concept of interest. Qualitative policy analysis is also a systematic process that aims to identify key concepts and themes. For example, Fisher et al. [22] undertook a qualitative analysis of health policy documents to determine the extent to which they address the social determinants of health and health inequities.

We undertook a review and analysis of six policy documents between August and November 2017. We selected health literacy policies from Australia, Austria, China, New Zealand, Scotland, and the United States for both practical and methodological reasons. Firstly, they were publicly available at the time of review and published in either English or German. Secondly, they were developed and implemented in diverse contexts, reflecting varying political and economic systems and conditions, and different healthcare systems, structures and public health approaches. Thirdly, the countries are sited in three WHO Regional groupings (Western Pacific, Europe and the Americas).

We adapted the policy analysis framework developed by Trezona et al. [23] to guide a systematic qualitative analysis of the six policy documents in this study. The authors developed the framework specifically for the purpose of analyzing the way in which policy documents prioritize, operationalize and resource health literacy. They then tested the utility of the framework by applying it to a set of Australian health policies. The framework has been tested and shown to be useful for analyzing policy documents containing health literacy, thus we determined it to be suitable for this study. However, the framework was previously applied quantitatively to rate and rank policy documents. We considered a qualitative approach to be more appropriate for achieving the aim of this study, therefore we adopted a number of criteria from the framework to guide a qualitative analysis and data extraction process. These were the criteria relating to policy objectives or priorities, proposed actions and interventions, allocation of resources and intentions to monitor policy outcomes. We also added criteria relating to the policy context and target audiences. The full list of analysis criteria is shown in Table 1.

The authors undertook the first round of analysis by extracting data from the six policy documents and organizing it in data collection sheets according to the above criteria. In order to verify the accuracy of the data extraction and interpretation, we invited key informants from the six countries to comment on the data presented in the data collection sheets. Specifically, they were asked to comment on whether the findings had been misinterpreted or misrepresented, or if there was critical information missing from the results. We received feedback from five of the six countries (we were unable to get feedback from our key informant in New Zealand). This feedback was generally positive regarding the accuracy of the representation of the policy, and our interpretation of its key elements. It also enabled us to improve the accuracy of our analysis by reporting information that was not explicitly stated in the policy documents, such as governance and oversight responsibilities, sources and level of expenditure related to the policy, and monitoring and evaluation mechanisms that are in place. We have also made specific references to information provided by key informants where this was not verifiable through currently accessible documents.

We present case studies for each country to provide an overview of their policy approach and observations about their strengths and weaknesses. We then compare and contrast the policies to describe the similarities and differences between the objectives and actions described, and to reveal trends in approaches to health literacy policy development across countries.

## 3. Results

A descriptive summary of each country’s policy is presented below. This includes the policy title, year of publication and publishing organization, a brief description of the context in which the policy was developed, and an overview of the high-level policy priorities, objectives, and/or action areas.

### 3.1. Australia

Federal, state and territory and local governments have a shared responsibility for health governance in Australia, including policy development and implementation, and the management of healthcare systems. Their respective roles are specified in the National Healthcare Agreement [24] The federal government has responsibility for the three core elements of Australia’s universal public health system: (i) The national public health insurance scheme (Medicare), which provides free or subsidized benefits for most medical, diagnostic and allied health services; (ii) The Pharmaceutical Benefits Scheme (PBS) which provides subsidized prescription medications; and (iii) The private health insurance rebate, which covers private hospital services and many out-of-hospital services not covered by Medicare [25]. State and territory governments are primarily responsible for public hospitals, ambulance services, community health services and mental healthcare.

The federal government is responsible for developing policies that set a national public health agenda, whereas state and territory governments set the public health agenda for their jurisdictions, as well as develop the program and funding guidelines that mandate the way services are expected to operate, including specific targets for service delivery. National advisory and regulatory bodies also play a significant role in shaping the priorities and direction of healthcare in Australia, and in monitoring the performance of the healthcare system.

In 2014 the *National Statement on Health Literacy* was released in Australia [26]. The Australian Commission on Safety and Quality in Healthcare (ACSQHC) is a corporate Commonwealth entity, jointly funded by the federal and state and territory governments in Australia. Its role is to ensure safe and high-quality health systems, including through the establishment of the National Safety and Quality Health Service Standards [27] and the ongoing accreditation of healthcare services. The ACSQHC is not a governing body, therefore the National Statement does not constitute a formal government policy, however it was endorsed by all federal, state and territory health ministers, signaling their at least in-principle commitment to addressing health literacy in Australia.

The ACSQHC developed the National Statement in order to increase an understanding of health literacy across relevant sectors, and promote a coordinated and collaborative approach to systematically addressing it nationally [26]. The document describes three strategic areas: (i) Embedding health literacy into systems; (ii) Ensuring effective communication; and (iii) Integrating health literacy into education. It also lists a range of actions divided into the role of consumers, healthcare providers, healthcare organizations, government organizations and policymakers (including regulatory and advisory bodies). The National Statement is reinforced by the incorporation of health literacy into the National Safety and Quality Health Service Standards. These standards are reviewed and revised regularly in consultation with the Australian Government, state and territory partners, consumers, the private sector and other stakeholders, and endorsed by all health ministers in Australia [28].

### 3.2. Austria

Responsibility for healthcare governance in Austria is shared between a number of institutions at federal and provincial levels. The federal government plays a central role in the development of legislation, with many implementation responsibilities delegated to provincial governments and social security institutions. Public health services are jointly provided by federal, provincial and local authorities, and supported by a compulsory, universal health and social insurance system. Ten national health targets currently guide healthcare reform in Austria to ensure coordinated planning, implementation and cross-sectoral action [29].

Health literacy is one of ten health targets for Austria, which were drafted in 2012 at the direction of the Federal Health Commission of Austria and the Austrian Council of Ministers [30]. In 2014 the Ministry of Health released the policy *National Health Target No. 3: Improving Population Health Literacy.* The policy is now being implemented in a two-fold approach, in which the aspects relating to the healthcare field are being implemented through the ongoing healthcare reform process, for which the Austrian Ministry of Health, the Austrian ‘Länder’ (Federal subdivisions) and social insurance have shared responsibility. The aspects relating to the ‘health in all policies’ dimensions of health literacy are being implemented through the intersectoral Austrian Health Literacy Platform [30].

The policy aims to improve health literacy for all people living in Austria, with a specific focus on vulnerable populations and sub-policies targeted to specific groups. The document describes three priority areas (articulated as sub-policies): (i) improve the health literacy-friendliness of healthcare services; (ii) improve individual health literacy (especially vulnerable groups); and (iii) improve health literacy-friendliness of the production and service sector. Actions under priority one include the implementation of a national strategy on improving the quality of communication in healthcare, promotion of the application of standard criteria (15 indicators) for good quality health information, the development of Austrian web portal on trustworthy health information, and making low threshold health consultation available through a 24-h telephone-based consultation service. Actions under priority two include health literacy-coaching for clients of health insurance companies, strengthening the responsibility of the education sector to lay the foundations for health literacy in schools, and supporting healthcare interactions of people who speak German (mother tongue) as a second language through a video interpreting service. Actions have not been articulated for priority three. However, it will focus on making it easier for consumers to make healthy decisions by providing good quality consumer information on products [30].

### 3.3. China

The Chinese National Government has overall responsibility for health legislation, policy development and administration in China, with local governments (provinces, cities, counties and towns) responsible for providing healthcare services. The National People’s Congress is responsible for health legislation, but policies may also be implemented by the State Council and Central Committee of the Communist Party. The National Health and Family Planning Commission (at the federal and local level) oversees healthcare delivery, including quality and safety, and administration of the Center for Disease Control and Prevention. In terms of health system financing, China has a publicly funded health insurance scheme, which is financed and provided by local governments and covers primary, specialist, emergency department, hospital care, mental healthcare, prescription and traditional medicine. The public health insurance scheme is complemented by a rapidly growing private health insurance scheme [31].

China released its *National Plan of Health Literacy Promotion Initiatives* for Chinese Citizens in 2008, which together with the ‘Health Literacy for Chinese Citizens–Basic Knowledge and Skills Trial’ (combining initiatives for health professionals and urban and rural residents) represents the Ministry of Health of the People’s Republic of China’s commitment to addressing health literacy, as well as the guiding document for the implementation of health literacy initiatives by all Provincial Departments of Health, autonomous regions, municipalities, Xinjiang Production and Construction Corps, the cities specifically designated in the State Plan, and the Chinese Center for Disease Control and Prevention [32].

The first part of the document outlines the objectives, targets and measures for the policy, and sets out the governance and accountability expectations for implementation of the policy by administrative departments at county, city and district levels. The second part of the document summarizes a range of content topics, divided into three sections: (i) basic knowledge and concepts; (ii) healthy lifestyles and behaviors; and (iii) basic skills, which presumably form the topics to be included in initiatives implemented at local levels.

The National Plan was developed for the purpose of promoting and popularizing the ‘Health Literacy for Chinese Citizens-Basic knowledge and skills Trial’, with four key objectives or targets:

1. To establish a health sector leading, multi-sector social participatory working-network of Health Literacy Promotion Initiatives with a coverage rate of 100 percent, 80 percent, and 60 percent in province, city/prefecture and county level respectively.
2. At least 80 percent of the professionals in the working-network to be trained.
3. To establish a Chinese citizens’ health literacy surveillance and evaluation system.
4. At least 60 percent of counties all over the country will carry out communication activities of ‘Health Literacy 66’ (the basic health knowledge and skills that have been identified for all Chinese citizens) [32].

The document also describes five key action areas or ‘tasks’ to be implemented as part of the policy, which can be briefly summarized as establish and improve the working network (leadership and governance), strengthen capacity building activities (workforce development), conduct communication activities, carry out health literacy surveillance, and develop demonstrative models (demonstration sites).

### 3.4. New Zealand

In New Zealand, the health system is largely publicly funded with the federal government responsible for setting the health policy agenda, service requirements and public funding allocation, while geographically defined district health boards are responsible for planning and delivering services. The Ministry of Health has overall responsibility for the health and disability systems, with support from specific purpose agencies such as the Health Promotion Agency and The Health Quality and Safety Agency [33].

While New Zealand does not have a specific health literacy policy, health literacy is incorporated as a key priority in the *New Zealand Health Strategy 2016–2026* [29]. The Strategy is required by New Zealand legislation that governs publicly funded health and disability services, which together with the New Zealand Disability Strategy, provides a framework for the delivery of all health and disability services. The Health Strategy is comprised of two documents; the main document establishes the broad ‘future directions’ for the health system and the companion document details the ‘roadmap of actions’ [34].

Health literacy is incorporated into the ‘people-powered theme’, one of five strategic themes within the strategy. The theme promotes building health literacy and active two-way engagement, with a broad focus on ensuring people have access to and can understand the information they need to manage their care, enabling individuals to make choices about their care, partnering with people to design services based on their needs and preferences, and communicating effectively and providing navigation support. Two action areas specifically relating to health literacy are described, one that focuses on individuals by providing health information to support self-management (including the use of digital technologies), and one that focuses on making the health system more health literacy responsive [35].

### 3.5. Scotland

The Scottish Government is responsible for healthcare legislation and financing, as well as setting national objectives and priorities for the National Health Service (NHS). NHS Health Boards (there are 14 across Scotland) are responsible for delivering health services that achieve the national objectives and priorities, which includes planning and commissioning hospital and community health services. The NHS Health Boards establish community health partnerships at a local level to ensure local authorities and patients are involved in healthcare delivery [36].

The Scottish Government released its first health literacy policy in 2014 with *Making it Easy: A Health Literacy Action Plan for Scotland*, the aim of which was for health and social care services to systematically address health literacy as a priority to improve health and reduce health inequalities, and for all people in Scotland to have the confidence, knowledge, understanding and skills needed to live well [37]. The Action Plan formed part of a broader policy agenda, contributing to the goals of Scotland’s 2020 Vision for Health and Social Care Policy, the NHS Scotland Healthcare Quality Strategy, and upholding the Patient Rights Act.

The first Action Plan was comprised of four key actions areas: (i) Increase workforce awareness and capabilities to address health literacy; (ii) Promote the development and spread of existing and new health literacy tools, innovations and technologies; (iii) Develop a National Health Literacy Resource including a ‘clearinghouse’ and community of practice; (iv) Establish a National Demonstrator Site.

Building on the progress made with the first action plan, the Scottish Government recently released its second action plan *Making it Easier: A Health Literacy Action Plan for Scotland 2017–2025* [38], which is a key element of its agenda to better support people through shared decision making. Its aim is to improve health literacy practice across the health and care system by removing barriers, making services easier to navigate, making information more responsive to need, and ensuring the design of services are informed by health literacy needs.

The second action plan describes three overarching action areas: (i) Share the learning from Making it Easy; (ii) Embed ways to improve health literacy in policy and practice; and (iii) Shift the culture by developing more health literacy responsive organizations and communities.

### 3.6. United States

In the United States, the health system is funded through a mix of public and private financing mechanisms, with the majority of the population relying on private insurance. The Affordable Care Act (2010) has been a key reform, and aims to achieve universal coverage, greater affordability and higher quality care, and establishes a shared responsibility for health insurance provision between the government, employers and individuals. In terms of health system governance, the United States (U.S.) Department of Health and Human Services is the principal federal government agency responsible for healthcare in the U.S. It is made up of a number of agencies and institutions, including the Centers for Medicare and Medicade Services (which play a central role in administering aspects of the public insurance schemes), the Centre for Disease Control and Prevention, and the Agency for Healthcare Research and Quality [39].

The U.S. Department of Health and Human Services (Office of Disease Prevention and Health Promotion) released the *U.S. National Action Plan to Improve Health Literacy* in 2010 [40], which aims to stimulate a society-wide movement to create a health literate America. The Action Plan is built on a vision of a society in which everyone has access to accurate health information, person-centred health information and services are delivered and lifelong learning to promote good health is supported. The document describes seven goals for achieving this vision:

1. Develop and disseminate health and safety information that is accurate, accessible, and actionable
2. Promote changes in the healthcare system that improve health information, communication, informed decision making, and access to health services
3. Incorporate accurate, standards-based, and developmentally appropriate health and science information and curricula in child care and education through the university level
4. Support and expand local efforts to provide adult education, English language instruction, and culturally and linguistically appropriate health information services in the community
5. Build partnerships, develop guidance, and change policies
6. Increase basic research and the development, implementation, and evaluation of practices and interventions to improve health literacy
7. Increase the dissemination and use of evidence-based health literacy practices and interventions.

These goals provide the frame for action, with a set of strategies outlined under each goal (154 in total), grouped according to the stakeholders with a potential role in implementing them [39].

### 3.7. Data Analysis

Results of the data analysis processes are presented in Table 2. The ‘Priority Areas’ column includes information that was expressed in the policy document as a priority, goal, objective or strategic area. The ‘Actions/Strategies’ have been summarized into key themes identified within the policy document, rather than the full list of actions. Data were only included in ‘Funding Allocation’ and ‘Monitoring and Evaluation’ columns if these were explicitly stated in the policy document. Our key informants were able to provide us with additional information, which is discussed later.

## 4. Discussion

The very existence of this first group of national health literacy policies and national strategies indicates that governments in significantly different parts of the world have recognized the need to respond to the personal and societal challenges represented by inadequate health literacy in populations. It is too early in the cycle of implementation to make definitive observations about the impact of these policies on health literacy in each of the countries. At this early stage, our goal was to consider the policies against established criteria for describing and assessing a health policy and to make some early observations that may be useful to countries considering the development of similar policies or national strategies.

From our analysis, we can observe that these policies all have some common features. All represent a response (at least in part) to perceived deficiencies in the quality of patient communication and patient engagement in the healthcare system. These responses range from structured guidelines and standards for healthcare organizations (such as in Australia), to the more instrumental (such as the “initiatives” and “demonstration sites” identified in the China strategy) [32] through to more aspirational statements (such as those reflected in the U.S. strategy) [39]. Most present health literacy as a universal challenge (applying to all patients and/or communities), with some also identifying groups who are higher priorities (for example children and young people in the U.S. Strategy) and/or at greater risk (for example Austria) [30]. All recognize the importance of professional education in improving the quality of communication, with Australia, China and Scotland having explicit commitments.

Most policies and strategies recognize that the responsiveness of the health system to variations in patient health literacy needs to be improved. These make clear that organizational change is required, with the necessary action expressed in different forms, such as “embedding health literacy into systems’ (Australia), “making the health system more responsive” (New Zealand); “building organizational health literacy responsiveness” (Scotland), and “promoting changes in the healthcare system” (U.S.).

China, the United States and to some extent Austria also have policies that overtly seek to influence and improve health literacy in the wider community, and identify a range of actions to support this. These range across improvements to adult literacy and language programs (U.S.), strengthening the role of the education sector in health literacy and providing better quality consumer information on products (Austria), through to more generic calls to public awareness of health literacy (Scotland).

Thus far, only China has undertaken population health surveys on an annual basis and these show modest but continuing gains in health knowledge and skills that are consistent with “Health Literacy 66” [32]. Of the other countries, Austria has baseline information on a population level from the European Health Literacy Survey [11], and Australia is undertaking a population survey in 2018 that might be used to monitor future change. The US has some data on some issues for some populations, but not universal population data on health literacy that is related to its national strategy.

Relative to the examples from China and Australia, the strategies in the U.S., New Zealand and Scotland appear to be more “enabling”, with less prescribed pathways for implementation, and variable mechanisms for monitoring and accountability. In Austria and New Zealand, the health literacy policy is embedded in a wider health and healthcare strategy, with some specific, funded activities, and a system for monitoring on a population level.

The other policies offer less obvious connections between objectives and practical actions. The U.S. has set clear targets for improving health literacy and has developed a comprehensive and integrated strategy to achieve those outcomes. But ownership and accountability are much less clear, and responsibility for action is diffuse. Some new resources are connected to the strategy, but not in an obvious and systematic form. The Scottish and New Zealand policies are clearly connected to the healthcare system with implied responsibility, but less well-defined ownership, accountability and resourcing.

Some strategies come with dedicated funding to support expected actions. China presents the clearest example of funding aligned to specific programs and activities, with Austria also identifying dedicated funding in support of the policy. The others are not systematically linked to new or dedicated funding, reflecting an underlying expectation that existing resources will be used differently or repurposed to support the ambitions of the policy. In the U.S. resources have been allocated to support some elements of the strategy, for example through enhanced funding for health literacy research.

As “first movers” in the development of national health literacy policies, the governments of these six countries are to be commended. These are all important public statements of priorities by government that have actual or implied commitment of resources and provide a mechanism for public accountability. When examined against established criteria for describing and assessing a policy some significant variations can be observed.

Based on publicly accessible information, the policy from China appears to be the most complete in having clearly defined goals, linked strategies and actions for different levels of government, some dedicated resources, and a system for monitoring and accountability. Australia has a highly structured approach to working with healthcare services based on an established system for monitoring health system standards and backed by an accreditation process, but this approach is inevitably limited in its reach and impact with the wider population. Austria has a more complete and detailed national strategy, some commitment of resources that are linked to identified priorities, but not such a clear system for monitoring progress and accountability against the stated priorities. The U.S. has an impressively comprehensive strategy but less obvious systems for linking resources and defined action to the achievement of the strategy. Scotland and New Zealand both have approaches that are embedded within the health system but recognize the need for action beyond healthcare. Both are commendably enabling and aspirational, but currently lack detail on resourcing, monitoring and accountability.

These differences undoubtedly reflect the significant differences in healthcare systems, and in political preferences. For example, the contrast in approach taken by China and the U.S. reflects the significant differences in political and social structures in those countries, and the healthcare systems that emerge from such diversity. Australia, Austria, Scotland, and New Zealand have different forms of universal healthcare delivered through devolved governance structures. These health systems require significant but variable direct government investment and tend to be responsive to policies that are more enabling than prescriptive, more devolved than centralized.

### Limitations

In deciding on which policy statements to include in the review we were inevitably constrained by the availability of sufficient English and German language material to be able to review and make comparative observations. The structure that we used to examine the available information assisted us in making comparative observations but inevitably restricted our ability to reflect the fundamentally different social and political context in which policy is formed and acted upon. We were able to obtain some of this feedback by using “key informants” in each of the countries and have tried to add this to the discussion, but inevitably some of the subtle and nuanced differences between countries and their political systems are likely to have been lost in this process. This is most obvious when considering the tools and mechanisms for national policy implementation that vary so markedly between countries. Those countries with significantly devolved decision-making and relatively limited national policy implementation incentives (such as the U.S.), contrast markedly with countries having more centralized policy-making and well-defined structures for local implementation (such as China).

The increasing recognition of the potential for health literacy to promote health and improve health services/systems is driving the emergence of new policies all the time. We are aware that since completing this review, policies have been issued in Germany, and are under development in Belgium, the Czech Republic, the Russian Federation and Portugal, making this review both topical and timely [16].

## 5. Conclusions

This first group of policy statements on health literacy provide a rich resource for current analysis and future learning. Each can be assessed against objective criteria but must also be understood as highly context specific. There are many positives in all of the examples we have examined, particularly in respect of the public acknowledgement of the challenge to improve health literacy, the priority given to the responsiveness of the health system, and the stimulus to improve the education and training of front-line staff in the health system (and beyond).

Policies focused on improving the effectiveness of health services carry strong political weight, in promising improved clinical quality and safety, and better health outcomes for patients. A broader focus on developing health literacy through community health education was harder to find but has greater potential to develop health literacy skills with wider application across the life-course. The absence of a clear, substantial and practical commitment to building health literacy in community populations was a notable deficit in most of the policies. Both approaches are necessary to advance health literacy in populations.

There was significant variability in linking resources to specific strategies and actions, in the systems for monitoring progress, and in accountability for progress. This variability reflects some of the important contextual differences between countries and health systems. However, this lack of specificity will often have an impact on the priority given to improving health literacy relative to other, more “urgent” and politically higher-profile priorities that have more immediate and visible impact on healthcare delivery (for example reducing patient waiting times). This, in turn, can have an impact on the long-term sustainability of defined actions to improve health literacy in populations as its visibility slips.

## Figures and Tables

**Table 1:** Criteria for analyzing and extracting data from policy documents.

1	What is the name of the policy?
2	When was it published?
3	Who published the policy (i.e., government department)?
4	What was the policy context in which it was developed?
5	Who are the intended target audiences of the policy (i.e., consumers, health professionals, hospitals)?
6	What are the stated objectives or priority areas of the policy?
7	What are the stated actions/interventions/strategies for addressing health literacy?
8	Does the document explicitly state the allocation of funding to support policy implementation?
9	Does the policy explicitly state a commitment to monitoring/measuring the policy outcomes? If so, what monitoring/measurement mechanisms are proposed?

**Table 2:** Analysis of selected policy documents.

Country	Target Audiences	Priority Areas #	Actions/Strategies	Funding Allocation *	Monitoring & Evaluation (M&E) *
Australia	Healthcare providers Healthcare organizations Organizations that support healthcare workers Government organizations Education providers Social services	Embedding health literacy into systems Ensuring effective communication Integrating health literacy into education	Increase consumer knowledge and skills through education Improve the skills and practices of the health workforce Provide and support professional development Develop and implement health literacy policies (organization level) Improve healthcare design and delivery Advocate for funding allocation for health literacy initiatives	Not explicitly stated in the policy	Not explicitly stated in the policy.
Austria	People living in Austria Vulnerable groups	Improve the health literacy-friendliness of healthcare services Improve individual health literacy (especially vulnerable groups) Improve health literacy-friendliness of the production and service sector	National strategy to improve quality of communication in healthcare Apply standard criteria when developing health information Develop web portal Provide telephone-based consultation service Health literacy coaching for clients of health insurance companies, strengthen role of education sector in health literacy Provide video interpreting service Provide good quality consumer information on products	Not explicitly stated in the policy	Yes, in line with monitoring of all ten Austrian Health Targets. Includes monitoring progress intervention implementation and regular analysis of defined indicators. Will include use of the health literacy survey scheduled for 2019.
China	Health professionals Health organizations and health education institutes Autonomous regions and municipalities Urban and rural residents Chinese citizens across the whole country	To establish a health sector leading, multi-sector social participatory working-network of Health Literacy Promotion Initiatives with a coverage rate of 100 percent, 80 percent, and 60 percent in province, city/prefecture and county level respectively At least 80 percent of the professionals in the working-network to be trained To establish a Chinese citizens’ health literacy surveillance and evaluation system At least 60 percent of counties all over the country will carry out communication activities of ‘Health Literacy 66′	Establish and improve the working network (leadership and governance) Strengthen capacity building activities (workforce development) Conduct communication activities Carry out health literacy surveillance Develop demonstrative models (demonstration sites)	Yes. Centrally funded through government, including for related initiatives (Health Literacy 66 and the Basic knowledge and skills trial). Health administrative departments at all levels are also required to incorporate local Health Literacy Promotion Initiatives into the health sector’s annual budget to ensure their implementation.	Yes. Ministry of Health conducts monitoring and evaluation of the “Health Literacy Promotion Initiatives” to assess policy implementation. Autonomous regions and municipalities health administrative departments are required to undertake M&E according to the local conditions. The policy includes actions to set up M&E capability, including systems, technical support and training for professionals. A health literacy ‘surveillance’ system has been implemented.
New Zealand	Government agencies National bodies; i.e., Health Quality and Safety Commission New Zealand Maori and Pacific health providers District health boards Primary healthcare services Disability services Social care services	People have access to and can understand the information they need to manage their care Enable individuals to make choices about their care Partner with people to design services based on their needs and preferences Communicate effectively and providing navigation support	Support self-management of health through the use of digital technologies, such as social media, mobile applications and video games (7 sub-actions) Make the health system more responsive, including through shared decision making, cultural competence and increased engagement (7 sub-actions)	Not explicitly stated in the policy	Yes, the policy states that the Ministry of Health will monitor work undertaken on the actions in the roadmap. It states that a set of measures on health outcomes and equity will be used to track progress, however these are not contained within the document.
Scotland	People working in health and social care	Remove barriers (to access) Make services easier to navigate Making information more responsive to needs Ensure the design of services is informed by health literacy needs	Public awareness of health literacy Use of health literacy tools and techniques by professionals providing care Provide better information at point of entry and discharge from services Shared care planning and support Promote a culture of shared decision-making Support way-finding and navigation Build networks of health literacy champions Build the skills of the workforce (health and social care) Build organizational health literacy responsiveness (undertake organizational assessments)	Not explicitly stated in the policy	Not explicitly stated in the policy.
United States	Professionals Public and private sector organizations Communities Policymakers Educators Librarians Clinicians Social services providers Researchers	Develop and disseminate health and safety information that is accurate, accessible, and actionable Promote changes in the healthcare system that improve health information, communication, informed decision making, and access to health services Incorporate accurate, standards-based, and developmentally appropriate health and science information and curricula in child care and education through the university level Support and expand local efforts to provide adult education, English language instruction, and culturally and linguistically appropriate health information services in the community Build partnerships, develop guidance, and change policies Increase basic research and the development, implementation, and evaluation of practices and interventions to improve health literacy Increase the dissemination and use of evidence-based health literacy practices and interventions	The action areas of the plan align with the goals, with 158 strategies listed across the 7 action areas, summarized as: Develop and distribute high quality, accessible and culturally appropriate health information Improve health information, communication, and decisions making in the healthcare environment and improve access to health services Incorporate health information into child care and education curricula Provide adult education, English language programs, and culturally appropriate services in the community Build partnerships and develop guidelines and policies that support and promote health literacy Increase research and evaluation on interventions to improve health literacy Increase the uptake of evidence-based health literacy practices and interventions	Not explicitly stated in the policy	Not explicitly stated in the policy.

* Key informants provided useful additional information regarding funding and monitoring/evaluation, which is verifiable but not evident from the published document. We refer to this in the discussion. # ‘Priority Areas’ includes information that was expressed in the policy document as a priority, goal, objective or strategic area.
